# Assessing the Antioxidant Properties of *Larrea tridentata* Extract as a Potential Molecular Therapy against Oxidative Stress

**DOI:** 10.3390/molecules23071826

**Published:** 2018-07-23

**Authors:** Rachid Skouta, Karla Morán-Santibañez, Carlos A. Valenzuela, Abimael H. Vasquez, Karine Fenelon

**Affiliations:** 1 Department of Biology, University of Massachusetts, Amherst, MA 01003-9297, USA; 2 Department of Chemistry and Biochemistry, Border Biomedical Research Center, The University of Texas at El Paso, El Paso, TX 79968, USA; ksmoransant@utep.edu (K.M.-S.); ahvasquez@miners.utep.edu (A.H.V.); 3 Department of Biology, Border Biomedical Research Center, The University of Texas at El Paso, El Paso, TX 79968, USA; cavalenzuela4@miners.utep.edu

**Keywords:** oxidative stress, *Larrea tridentata* extract, antioxidant in vitro assays, cytotoxicity assay

## Abstract

Oxidative stress has been linked to neurodegenerative diseases such as Huntington’s, Parkinson’s, Alzheimer’s and amyotrophic lateral sclerosis diseases. *Larrea tridentata* (LT) also known as Creosote Bush is an evergreen shrub found in the Chihuahuan desert which has been used medicinally by Native American tribes in southwestern North America and the Amerindians of South America. However, studies of the antioxidant capacity of the crude extract of LT towards the discovery of novel molecular therapies bearing antioxidants and drug-like properties are lacking. In this study, we assessed the antioxidant properties of *Larrea tridentata*, collected specifically from the Chihuahuan desert in the region of El Paso del Norte, TX, USA. LT phytochemicals were obtained from three different extracts (ethanol; ethanol: water (60:40) and water). Then the extracts were evaluated in eight different assays (DPPH, ABTS, superoxide; FRAP activity, nitric oxide, phenolic content, UV visible absorption and cytotoxicity in non-cancerous HS27 cells). The three extracts were not affecting the HS27 cells at concentrations up to 120 µg/mL. Among the three extracts, we found that the mixture of ethanol: water (60:40) LT extract has the most efficient antioxidant properties (IC_50_ (DPPH at 30 min) = 111.7 ± 3.8 μg/mL; IC_50_ (ABTS) = 8.49 ± 2.28 μg/mL; IC_50_ (superoxide) = 0.43 ± 0.17 μg/mL; IC_50_ (NO) = 230.4 ± 130.4 μg/mL; and the highest phenolic content was estimated to 212.46 ± 7.05 mg GAE/L). In addition, there was a strong correlation between phenolic content and the free-radical scavenging activity assays. HPLC-MS study identified nine compounds from the LT-ethanol: water extract including Justicidin B and Beta peltain have been previously reported as secondary metabolites of *Larrea tridentata*.

## 1. Introduction

Radical species including reactive oxygen species (ROS) and reactive nitrogen species (RNS) have been linked to oxidative stress and neurodegenerative diseases such as Huntington’s, Parkinson’s, Alzheimer’s and amyotrophic lateral sclerosis [1]. Free radical species normally exist in all aerobic cells in balance with antioxidants (also known as scavengers or reducing agents) [2]. On the other hand, oxidative stress occurs when this critical balance is disrupted under depletion of free radicals, antioxidants or both [3]. Various endogenous (e.g., mitochondrial respiratory chain, cytochrome 450) and exogeneous (e.g., air pollutants, smoke, and pesticides, or ultraviolet light) factors have been shown to lead to an excessive free radical species and oxidative stress [4,5].

In addition, free radicals are highly reactive chemical species that contain one or more unstable unpaired electrons. These unpaired electrons are continuously produced in the human body due to their various functions such as chemical signaling, energy supply, detoxification, and immune function. However, excess of these unstable unpaired electrons is likely altering several molecules (e.g., proteins, RNA, DNA, and lipids) in the human body, through the known radical propagation pathways (initiation, propagation and termination). For example, it is well known that the reduction of molecular oxygen generates ROS species (superoxide anion radical (O_2_–•), singlet oxygen (1O_2_), hydrogen peroxide (H_2_O_2_), lipid peroxides (R–O–OH) and the highly reactive hydroxyl radical (•OH). On the other hand, RNS are mainly derived from the reduction of nitrate (NO_3_–) and nitrite (NO_2_–) to nitric oxide (NO_x_) [6,7]. Therefore, identifying new therapeutic strategies capable of modifying the course of oxidative stress and neurodegenerative diseases are currently of major interest [8,9,10]. Moreover, the discovery of novel antioxidant compounds bearing better pharmaceutical properties (e.g., better solubility, bioavailability and stability while no significant toxicity) is a major thrust area [11].

We previously reported the ability of a synthetic antioxidant arylalkyl amines to prevent various forms of cell death involving lipid peroxidation such as Huntington’s disease, periventricular leukomalacia, and kidney dysfunction [12,13,14,15]. Antioxidants such as diarylamines, butylated hydroxytoluene (BHT) and butylated hydroxyanisole (BHA) are used in the food industry to prevent spoilage [16]. However, the toxicity studies of these synthetic antioxidants such as butylated hydroxytoluene (BHT) and butylated hydroxyanisole (BHA) have an impact on human health [2]. Thus, a replacement of the synthetic compounds by phenolic antioxidant extractions from various foods and plants has been proposed [17].

Recently, interest has been growing in the search for potent plants-based phytochemicals with antioxidant properties towards the development of molecular combined therapies and alternative medicine [18,19]. *Larrea tridentata* (LT) is an evergreen shrubby plant belonging to Zygophyllacea family [20]. It is also known as Creosote Bush, chaparral or greasewood. LT dominates the Chihuahuan desert and highly used in traditional medicine by the native population of Mexico [21]. LT were known by their pharmacological activities such as antiherpes, antioxidant, antifungal, and anti-inflammatory. In addition, they were used against human immunodeficiency virus, human papillomavirus, cancer, neurodegenerative diseases, and symptoms of aging. A thorough reviews of the medicinal uses of LT was published by Arteaga et al. in 2005 and Gnabre et al. in 2015 [22,23].

It is worthwhile mentioning a recent study reported, in 2016, by Del Vecchyo-Tenorio et al. regarding the efficacy of LT against obesity, which is still remaining one of the major health issues worldwide. More specifically, the authors reported that the addition of LT ethanolic extract to standard diet in hampsters reduced high fat, plasma glucose, cholesterol, and peroxidation, and increased sensitivity to insulin. Their study suggested that the ethanolic extract of LT could be useful in the treatment of type 2 diabetes [24]. Recently, Bashyal et al. reported that *Larrea tridentata* possess anti-parasitic activity against *Entamoeba histolytica*, *Giardia lamblia* and *Naegleria fowleri* [25].

**Figure 1:**
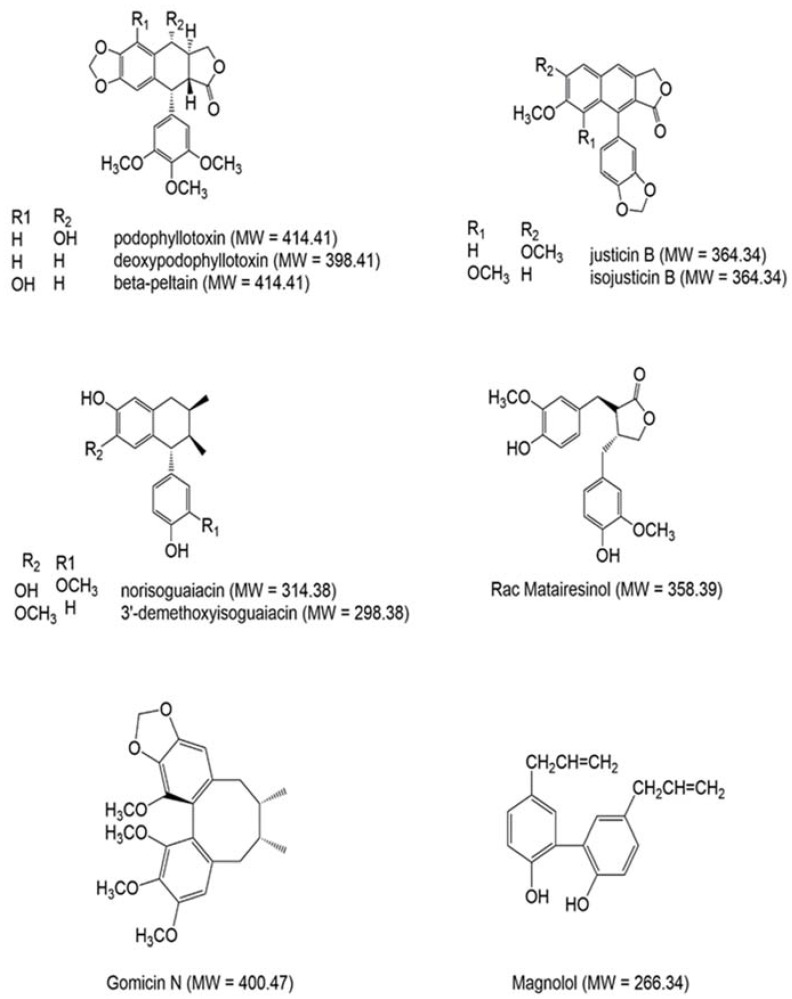
Example of selected natural products found in *Larrea tridentata* (LT) plant.

The nordihydroguaiaretic acid (NDGA) is the most abundant and studied metabolite of LT [22]. NDGA is a potent inhibitor of lipoxygenase and cyclooxygenase pathway [26]. Tea brewed of LT was used to treat digestive disorders, rheumatism and most common colds [23]. In 2018, Chan et al. reported that NDGA protect against obesity in vivo model [26]. LT is a rich source of metabolites including phenolic, terpenoids, saponins, and odorous vinyl ketones (Figure 1) [27]. Abundant alkaloids and phenolic compounds such as chlorogenic acid have been found in a methanol extract of *Larrea tridentata* (growing site not reported) [28]. It has been previously reported that the amount and type of phytochemicals can vary in the same plant. These variations are depending on various factors such as height, age, growing site and also agronomics and environmental conditions [29,30,31]. In this study, we used three solvents to extract phytochemicals from LT and investigated their antioxidant capacity, for the first time, in different in vitro antioxidant assays. The in vitro assays represent most of the known radical species generated under oxidative stress causing cancer and neurodegeneration. These assays could be effectively utilized to quantify the bioactive compounds for future phytopharmacological applications. Therefore, plants-based medicine would be an attractive source of natural drugs bearing antioxidants’ properties.

## 2. Results

### 2.1. LT Dried Leaf Extraction

We performed the LT dried leaves in three different solvents (ethanol, water, and ethanol: water (60:40 *v*:*v*) at 40 degrees Celsius for 4 h then at room temperature for 24 h. The ethanol and ethanol: water (60:40 *v*:*v*) extracts were collected as a dark brown oil, while the water extract was collected as a brownish solid (Table 1). As illustrated in Table 1, LT-ethanol extract weighed 3.144 g (yield, 19.65%), LT-ethanol: water (60:40 *v*:*v*) weighed 3.964 g (yield, 24.78%) and LT-water weighed 2.258 (yield, 14.11%).

### 2.2. DPPH (2,2-Diphenyl-1-picrylhydrazyl) Radical Scavenging Activity

**Figure 2:**
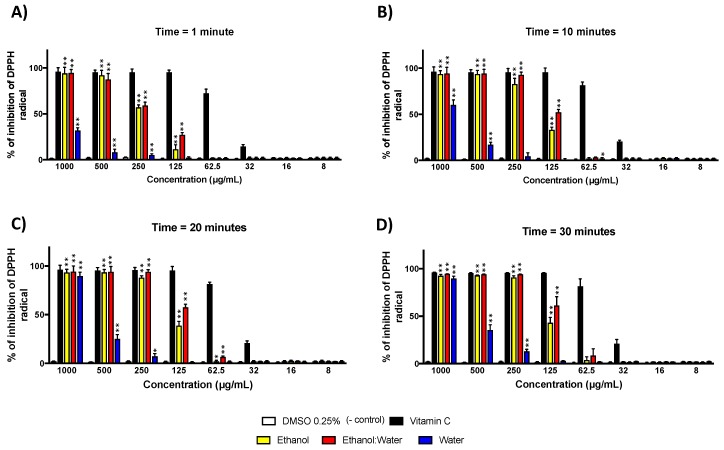
DPPH radical inhibition of three LT extracts at different concentrations (1000 μg/mL–8 μg/mL and at four time points: (**A**) time = 1 min, (**B**) time = 10 min, (**C**) time = 20 min and (**D**) time = 30 min. The concentration of the DPPH radical was 100 μM. The absorbance was measured at 517 nm. Each bar represents the average of three replicates and the error bars represent the standard deviation. The asterisks indicate a significant difference between the treatment and negative control (* *p* < 0.05 and ** *p* < 0.001).

We performed the radical scavenging assay using the commercially available stable nitrogen radical species DPPH against the three LT plant extracts (ethanol, water and water: ethanol). To do so, we tested DPPH radical at 2 mM concentration against each extract at various concentrations (1000–8 μg/mL), and at several time points (1, 10, 20 and 30 min). The kinetic study showed a good correlation between the time of incubation and the radical scavenging activity of the three LT extracts (Figure 2). Moreover, LT-ethanol: water extract showed the most antioxidant scavenging activity (IC_50_ at 30 min = 111.17 μg/mL) followed by LT-ethanol extract (IC_50_ at 30 min = 135.4 μg/mL) and then the LT-water extract (IC_50_ at 30 min = 572.7 μg/mL). DMSO and vitamin-C (ascorbic acid), the known antioxidant small molecule, were used as negative and positive controls, respectively (Figure 2).

### 2.3. ABTS (2,2′-Azino-bis(3-ethylbenzthiazoline-6-sulphonic acid)) Radical Scavenging Activity

**Figure 3:**
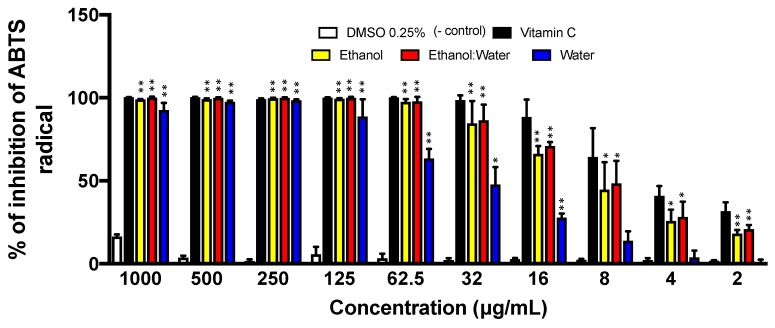
ABTS radical cation inhibition of three LT extracts at different concentrations (1000 μg/mL–2 μg/mL after 20 min incubation. The concentration of the ABTS was 100 μM. Each bar represents the average of three replicates and the error bars represent the standard deviation. The asterisks indicate a significant difference between the treatment and negative control (* *p* < 0.05 and ** *p* < 0.001).

We performed the ABTS radical scavenging activity of the three LT extracts in a similar way as the DPPH assay. The LT-ethanol: water extract showed the most radical scavenging activity with an IC_50_ of 8.49 μg/mL, followed by the LT-ethanol extract with an IC_50_ of 9.75 μg/mL. On the other hand, LT-water extract showed the lowest antioxidant scavenging activity with an IC_50_ of 35.84 μg/mL. DMSO and vitamin-C (ascorbic acid), the known antioxidant small molecule, were used as negative and positive controls, respectively (Figure 3).

### 2.4. Superoxide Radical Assay

**Figure 4:**
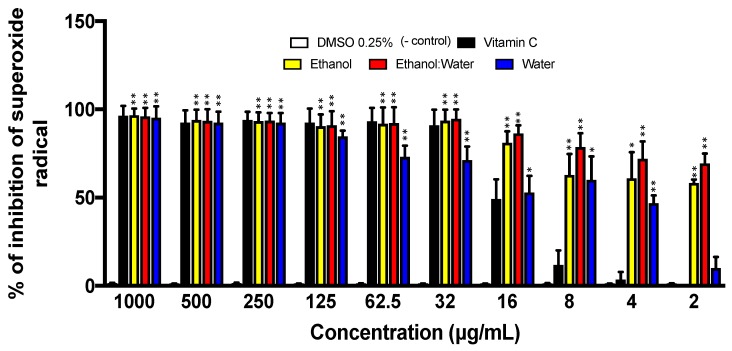
Superoxide scavenging activity of three LT extracts at different concentrations (1000 μg/mL–2 μg/mL) after 20 min incubation. Each bar represents the average of three replicates and the error bars represent the standard deviation. The asterisks indicate a significant difference between the treatment and negative control (* *p* < 0.05 and ** *p* < 0.001).

Superoxide radicals were generated in situ by the alkaline DMSO method to assess the scavenging activity of the three LT extracts. Overall, the ethanol: water-LT, ethanol-LT, and water-LT extracts strongly inhibited the superoxide radical generation at IC_50_ of 0.43 µg/mL, 2.1 µg/mL and 10.1 µg/mL, respectively. In addition, the three LT-extracts showed better efficacy in radical scavenging activity compared to vitamin-C. DMSO and vitamin-C, the known antioxidant small molecule, were used as negative and positive controls, respectively (Figure 4).

### 2.5. Nitric Oxide Scavenging Assay (NO) Using the Griess Reagent

**Figure 5:**
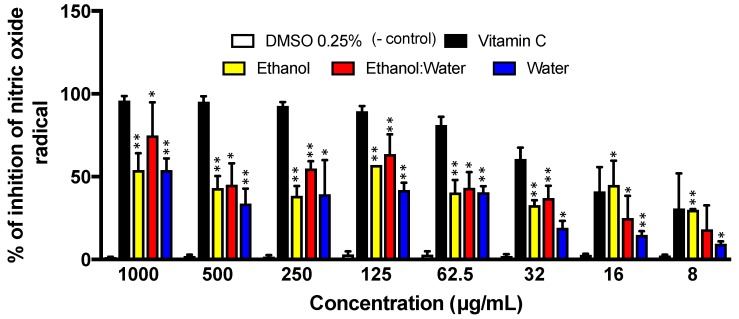
Nitric Oxide (NO) inhibition of 3 LT plant extracts at different concentrations (1000 μg/mL–8 μg/mL) after 4 h of incubation. The absorbance was recorded at 564 nm. Each bar represents the average of three replicates and the error bars represent the standard deviation. The asterisks indicate a significant difference between the treatment and negative control (* *p* < 0.05 and ** *p* < 0.001).

The three LT extracts showed a very modest inhibition of nitric oxide radical scavenging activity compared to the other radical species such as ABTS and superoxide radicals (Figure 3 and Figure 4). The LT-ethanol: water extract showed the most radical scavenging activity with an IC_50_ of 230.4 ± 130.4 μg/mL, followed by the LT-water extract showed the lowest antioxidant scavenging activity with an IC_50_ of 520.7 ± 100.4 μg/mL. On the other hand, LT-ethanol extract showed the lowed radical scavenging properties with an IC_50_ of 551.3 ± 112.4 μg/mL (Figure 5). DMSO and vitamin-C, the known antioxidant small molecule, were used as negative and positive controls, respectively (Figure 5).

### 2.6. Ferric Reducing Antioxidant Power Assay (FRAP)

**Figure 6:**
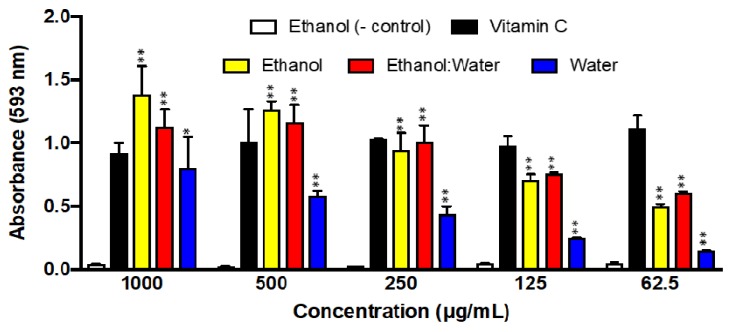
Iron Reducing Antioxidant Potential (FRAP) of three LT plant extracts at different concentrations (1000 μg/mL–62.5 μg/mL) after 5 min of incubation. The absorbance was recorded at 593 nm. Each bar represents the average of three replicates and the error bars represent the standard deviation. The asterisks indicate a significant difference between the treatment and negative control (* *p* < 0.05 and ** *p* < 0.001).

The reductive ability of the three LT extracts is shown in Figure 6. Overall, the LT-ethanol and LT-ethanol: water extracts showed at least one-fold better FRAP activity than LT-water extract. In addition, the ethanol-LT and ethanol: water-LT extracts showed better reductive potentials than vitamin C at high concentrations. DMSO and vitamin-C, the known antioxidant small molecule, were used as negative and positive controls, respectively (Figure 6).

### 2.7. Estimated Total Phenolic Content

We used the well-known Folin–Ciocalteu method to estimate the total phenolic content (TPC) of the three LT extracts (LT-ethanol, LT-ethanol: water and LT-water). The TPC of each extract was determined using a regression equation of the calibration curve (*y* = 13.33 + 0.0004, R^2^ = 0.9985) and expressed as gallic acid equivalents (GAE). Together, the TPC of LT-ethanol, LT-ethanol: water and LT-water were found to be 200.23 ± 6.25, 212.46 ± 7.05 and 115.06 ± 10.11 mg GAE/L, respectively (Table 2).

### 2.8. Ultraviolet (UV)-Visible Spectroscopy Assay

**Figure 7:**
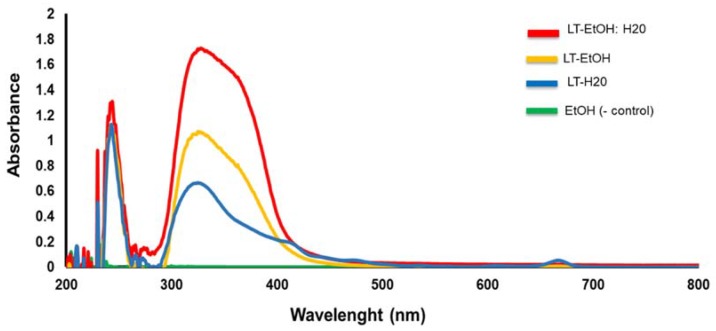
UV visible absorption analysis, between 200–800 nm, of LT-EtOH: H_2_O extract (red color); LT-EtOH extract (orange color) and LT-H_2_O extract (blue color). The absorption of EtOH alone (green color) was used as a negative (−) control.

We performed the UV-visible measurement of the three LT extracts (LT-ethanol, LT-ethanol: water and LT-water) (Figure 7). Each of the three extracts was scanned from the highest (800 nm) to the lowest (200 nm) wavelength. Overall, each of the three extracts showed a sharp absorption at 240 nm followed by a large absorption at 330 nm. In addition, the three extracts showed almost similar absorption intensity (AI = 1.1–1.3) at 240 nm wavelength. On the other hand, at 330 nm wavelength, the LT-ethanol: water extract showed the highest absorption intensity (AI = 1.7), followed by the LT-ethanol (AI = 1.1) and then the LT-water (AI = 0.6), respectively. Ethanol alone was used as a negative control (Figure 7).

### 2.9. Cytotoxic Activity of LT Extracts

**Figure 8:**
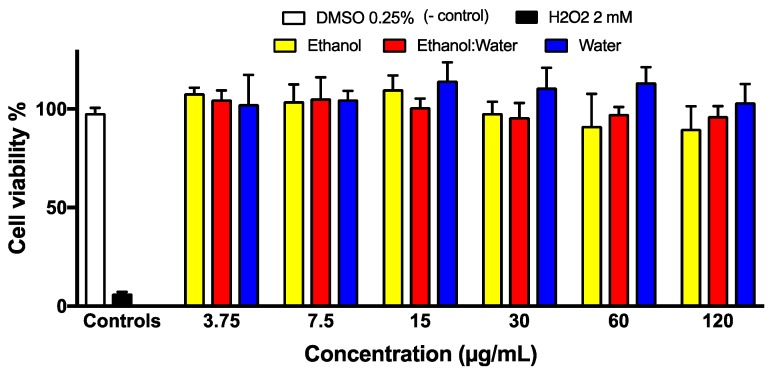
Cytotoxicity study of LT extracts against Hs27 cells. The non-cancerous Hs27 fibroblasts cells were incubated for 24 h after treatment with each of the three LT extracts at various concentrations (3.75–120 μM). DMSO and H_2_O_2_ were used as negative and positive controls, respectively. Each bar represents the average of three replicates.

The non-cancerous human skin fibroblasts Hs27 cells were exposed to different concentrations (3.79–120 μg/mL) of the three LT extracts (LT-ethanol, LT-ethanol: water and LT-water) at 24 h of incubation. DMSO at 0.25% and H_2_O_2_ at 2 mM, the known cytotoxic small molecule, were used as negative and positive controls, respectively (Figure 8). Based on our cell viability assay, the 3 LT extracts did not alter the HS27 cells and showed low cell death that is similar to DMSO, the negative control (Figure 8).

### 2.10. Phytochemical Identification by High Performance Liquid Chromatography (HPLC) and Mass (MS) Analysis

**Figure 9:**
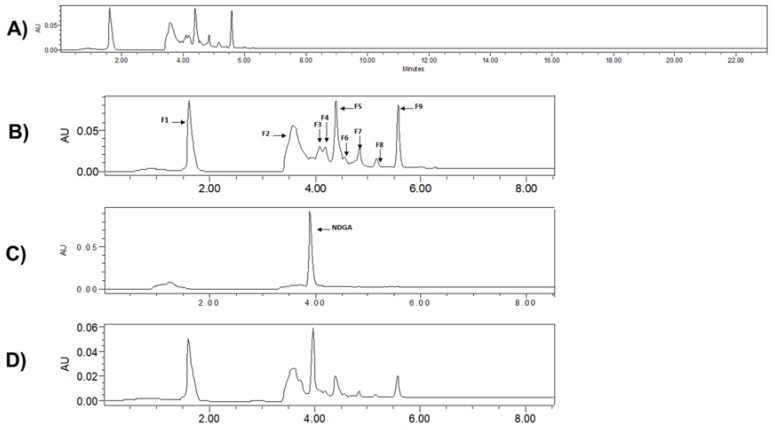
Chromatograms obtained for: (**A**) *Larrea tridentata* (LT)-ethanol: water extract alone at 24 min; (**B**) zoom in of *Larrea tridentata* (LT)-ethanol: fractions alone; (**C**) zoom in of nordihydroguaiaretic acid (NDGA) alone and (**D**) zoom in of mixture of LT-ethanol: water extract and NDGA as pure compound, detection at 280 nm. The fractions collected are numbered (F1–F9) as indicated.

We first optimized the HPLC conditions for the LT-ethanol: water extract using various injection volumes/concentrations at several flow rates (1.2 mL/min to 0.25 mL/min) and wavelengths (240 nm, 280 nm and 330 nm). We then used our optimized HPLC method (10 μL at 2.5 ppm, 0.5 mL/min, 280 nm to run LT-ethanol: water extract for 24 min to collect the fractions (Figure 9A). Nine potential fractions, as shown in Figure 9B, were collected and submitted to mass analysis. We finally used the most known mass spectral database MassBank [32], to identify the isolated fractions. The database enabled us to annotate unknown compounds with their structures, including chemical structure information [33]. The collected potential identified fractions from the LT-ethanol: water extract are shown in Table 3.

We also performed the HPLC of: (i) the known natural compound NDGA, in a pure form, which is the most abundant metabolite in LT (Figure 9C) and (ii) the mixture of LT-ethanol: water extract and NDGA as pure compound (Figure 9D). Overall, based on the chromatograms shown in Figure 9, it is likely that our LT-ethanol: water extract does not contain the most abundant metabolite (NDGA) in LT.

## 3. Discussion

In this study, we investigated the antioxidant activity of LT plant extracts in ethanol, ethanol: water (60:40), and water in various in vitro models that represent most of the free radical species involved in cancers and neurodegeneration diseases. Since free radicals are of different chemical entities involved in these diseases, it is essential to test the LT extracts against various types of in vitro generated free radicals to provide a general antioxidant activity profile of LT. We started our investigation with the DPPH assay, which is a nitrogen stable free radical (Figure 2). DPPH assay has been widely accepted as a tool for estimating free radical scavenging activities of antioxidants. Originally, it was believed that the DPPH assay involved a hydrogen transfer reaction. However, Foti et al. [34] reported another mechanism that consisted of two-step reactions. First, a quick electron transfer followed by a slow hydrogen transfer, which also depends on the hydrogen bond accepting solvent such as ethanol or methanol [35]. In our assay, the % of inhibition of DPPH radical was determined by the decrease in its absorbance at 517 nm [36]. Vitamin-C (ascorbic acid) is a common natural antioxidant compound found in many fruits and vegetables was used, in a pure form, as a positive control in our assays (Figure 2). The ethanol: water extract at 40 °C for 4 h proved to be the strongest radical scavenger in the DPPH assay, which was closely followed by ethanol, and then water extracts obtained at 40 °C for 4 h. These data suggested that the optimal potential antioxidants of *Larrea tridentata* (LT) are found to a mixture of ethanol: water solvents. The most abundant antioxidant in LT is NDGA, which is known to be slightly soluble in hot water [22]. However, this would not explain why the LT-ethanol: water performed better than LT-ethanol. If the scavenging activity was only due to NDGA both extracts would have been almost identical. In addition, since both extracts were the same weight, the percentage of NDGA in LT-ethanol should be higher than in LT-ethanol: water. Since, by using some water, other molecules would have been extracted, this could suggest that other antioxidant type scavengers are present in our LT-ethanol:water extract. It is also important to note that the steric effect of the DPPH radical is a major determinant of the reaction, which means that small molecules have relatively higher antioxidant capacity compared to larger and sterically hindered molecules [35].

The ABTS radical is another type of nitrogen cation radical scavenging assay (Figure 3). The ABTS radical scavenging assay involves a more drastic radical, which is chemically produced and often used for screening complex antioxidant mixtures such as plant extracts, beverages, and biological fluids [2]. This is due to its applicability in both aqueous and lipid phases [37]. Other advantages of using the ABTS assay are that it is easy, fast, leads to reproducible results, and eliminates color interference by plant extracts since the absorption wavelength is 734 nm [2]. The ABTS is a stable nitrogen radical cation, which has a blue-green chromosphere absorption produced by oxidation of ABTS with sodium persulfate prior to the addition of antioxidant [38]. The three LT-extracts showed good antioxidant activity in quenching the ABTS radical species. However, they lost efficacy quickly as the concentration drops compared to the control vitamin-C. Overall, the LT-ethanol: water extract showed the most potent antioxidant scavenging activity among the three LT extracts (Figure 3).

Superoxide radicals are known to be very harmful to cellular components as precursors of oxidative stress including reactive oxygen species [39]. In this experiment, the superoxide radical was created by the alkaline DMSO method, for which ethanol, and ethanol: water showed a strong inhibition of the radicals created, in fact stronger scavenging activity than the control, ascorbic acid. LT-water, showed the least antioxidant activity, slightly better than the control at its lowest concentration. The most potent radical scavenger was ethanol: water extract, which suggest that the most efficient extraction made was a mixture of ethanol: water (60:40) (Figure 4).

Nitric oxide molecules such as NO_2_, N_2_O_4_, N_3_O_4_ are very reactive compounds and could be formed during their reduction reaction in the presence of oxygen or superoxide to form nitric oxide radical types (e.g., NO•). These nitric oxide radical-types are produced in various cells, including brain cells, endothelial cells and macrophages [40]. Their toxicity could be due to the reaction of nitric oxide with superoxide (O•^2−^), which produces a powerful toxic oxidant peroxynitrite (ONOO^−^) [41]. The nitric oxide assay is a competitive assay for which the radical scavenger competes with molecular oxygen to react with the nitric oxide radical. In the present study, LT extracts offer a medium to poor scavenging of nitric oxide species, compared with the positive control, ascorbic acid (Figure 5).

Next, we measured the reducing ability of the three LT extracts using the reduction transformation of Fe^3+^ to Fe^2+^. Fe^3+^ reduction is often used as an indicator of electron donating activity, which is an important mechanism of phenolic antioxidant action and can be strongly correlated with other antioxidant properties [31]. Overall, the data of LT-ethanol: water extract correlates with the results obtained in the other in vitro assays (Table 2 and Table 4). LT-water showed the lowest reducing activity of Fe^3+^ to Fe^2+^ of all three extracts. On the other hand, LT-ethanol and LT-ethanol: water extracts have very similar reducing abilities, which correlate with DPPH, ABTS, and phenolic content (Table 2 and Table 4). This assay consists on reducing Fe^3+^ to Fe^2+^, since antioxidants are often reducing agents that prevent oxidative reactions. Once the Fe^+2^ is formed by the presence of a reducing agent, *O*-Phenanthroline monohydrate making a complex with the iron, and this complex can be measured at 510 nm. It is important to note that vitamin-C from concentrations 1000 µg/mL–125 µg/mL are lower than concentrations 62.5 µg/mL–32 µg/mL. This could be due to the fact that vitamin-C at high concentrations not only reduce, but also chelate the iron, thus disrupting the *O*-Phenanthroline monohydrate -Fe^2+^ complex (Figure 6).

Total Phenolic Content of the three LT-extracts was measured (Table 2). Among the three extracts, the LT-ethanol: water showed the best TPC of 212.46 ± 7.05, which correlates well with the best antioxidant properties that were shown in all radical scavenging assays (Table 2 and Table 4).

Toxicity studies of LT plant extracts were previously reported by various groups. For example, Aarland et al. demonstrated the low toxicity of a LT-methanolic extract in an in vivo model of *Artemia salina*, with a lethal dose fifty (DL_50_) of 1592 mg/mL [28], which means that the plant is less toxic in this in vivo model. Towards assessing the potential toxicity of LT-ethanol, LT-ethanol:water and LT-water extracts, we evaluated their cytotoxicity against a known non-cancerous Hs27 fibroblasts cells at different concentrantions, ranging from 3.75 μg/mL to 120 μg/mL in (Figure 8). Overall, no significant cell death was observed at concentrations up to 120 µg/mL compared to the negative control (DMSO 0.25%) treatment. In addition, cytotoxicity of LT extracts against various cancer cell lines has been reported previously. For example, Luo et al. determined the reduction on cell viability of a methanol extract on P388, a murine leukemia line with a EC_50_ of 0.57 µg/mL [42,43]. Based on our study and the reported literature, LT extracts exhibits high selective toward cancer cells while not affecting the non-cancerous cells.

UV-Vis, HPLC and mass analysis, respectively, allowed us to identify potential natural products. More specifically, nine fractions from the LT-ethanol:water extract were collected and identified in this study. Some of the tentative compounds were identified as Justicidin B and Beta peltain have been previously reported as secondary metabolites of *Larrea tridentata*. Overall, the majority of the tentative compounds are phenolic compounds with antioxidant activity that have been previously reported in other plants related to *Larrea tridentata* [44,45,46].

## 4. Materials and Methods

### 4.1. Chemicals

Folin–Ciocalteu’s phenol reagent (cat# 47742); 2,2′-azino-*bis*(3-ethylbenzthiazoline-6-sulphonic acid) (ABTS) (cat# 10102946001), *O*-Phenanthroline (cat# 516705) and the Griess reagent (cat# 516705) were purchased from Sigma (St Louis, MO, USA). Griess Reagent, ACROS Organics (cat# AC328670500); Sodium persulfate (cat# AC202025000) and 1, 10-Phenanthroline (cat# P69100) were purchased from Fisher Scientific (Pittsburgh, PA, USA). All purchased chemicals were used without further purifications.

### 4.2. Plant Material and Extract Preparation

Plant Material. *Larrea tridentata* (LT) leaves were collected from the Chihuahuan desert in the region of El Paso del Norte, TX, USA. The leaves were authenticated by a plant biodiversity expert at the University of Texas at El Paso (Professor Emeritus: Richard D. Worthington).

Plant extract preparation. In addition, 16 g of LT leaves were extracted in different solvents (ethanol, water, and ethanol: water [60:40 *v*:*v*]) at 40 degrees Celsius for 4 h then at room temperature for 24 h. The three extracts were filtered using Celite^®^ 545 to remove the leaf material. The solvents were smoothly evaporated under vaccum or lyophilizer at a low temperature. The extracts were stored at 4 °C until further use. The ethanol and ethanol: water (60:40 *v*:*v*) extracts were collected as a dark brown oil, while the water extract was collected as a brownish solid with reasonable yield (Table 1).

### 4.3. Radical Scavenging Assay Using the Stable Radical 2,2-Diphenyl-1-picrylhydrazyl (DPPH•)

We used our previously reported protocols [8,9] to perform this experiment with our LT extracts. A 2 mM stock solution of DPPH was prepared by dissolving 15.77 mg of DPPH• into 20 mL of methanol. The stock solutions of the three LT extracts were prepared by dissolving 38 mg of each extract in 1 mL of DMSO (Dimethyl sulfoxide) and filtered with a Millipore membrane filter (Bedford, MA, USA) (0.45 μm) prior to the radical inhibition testing. In addition, 10 µL from the stock solution of each extract was added to 190 µL of methanol for a final concentration of 2 mg/mL. In a 96-well microplate, a two-fold dilution of each extract at concentrations from 2000 µg/mL to 8 µg/mL was prepared at a total volume of 100 uL/well. Then, an equal volume of 100 µL of 2 mM of DPPH was added to each well. The final concentration of DPPH was 1 mM, and the final concentration of the samples ranged from 2 (µg)/mL to 1000 (µg)/mL. The LT extracts radical scavenging efficacy was measured immediately (1 min) after the addition of 2 mM DPPH then at 10, 20 and 30 min at 517 nm. Experiments were performed in triplicate. Equation (1) was used to calculate the percentage (%) of inhibition of the three LT extracts:

% of Inhibition = ((A_o_ − A_1_)/A_o_) × 100%, (1)

where A_o_ is the standard. A_1_ is the sample tested.

### 4.4. ABTS Radical Cation Assay Using 2,2′-Azino-bis(3-ethylbenzthiazoline-6-sulphonic acid)

The free radical scavenging capacity of several plant extracts has been widely studied using the 2,2′-azino-*bis*(3-ethylbenzthiazoline-6-sulphonic acid) radical cation (ABTS+•) decolorimetric assay, which is based on the reduction of the ABTS+• by antioxidants [17]. We used Dudonne et al. protocol [47] to perform this experiment with our LT extracts. (ABTS+•) was dissolved in deionized (DI) water to a concentration of 7 mM (72 mg of ABTS+• into 20 mL of DI water. A stock solution of sodium persulfate (Na_2_S_2_O_8_) of 2.45 mM was prepared by dissolving 11.7 mg of Na_2_S_2_O_8_ in 20 mL of DI water. These two sock solutions were mixed in a ratio of 1/3 ABTS to 2/3 Na_2_S_2_O_8_ which was made with 13.3 mL of sodium persulfate and 6.6 mL of ABTS. This solution was allowed to stand in a dark room at room temperature for 12–16 h, thus making ABTS•+. The final concentrations of sodium persulfate, and ABTS were 1.63 mM, and 2.31 mM, respectively. The stock solution of each extract and the control were prepared in DMSO to a concentration of 4.76 mg/mL. In addition, 43 μL of each extract was added to 157 μL of ethanol to a final volume of 200 μL, and then 100 μL was taken and dissolved in 100 μL of ethanol, in order to achieve a two-fold dilution. The sample concentrations ranged from 2 μg/mL to 1000 μg/mL. Then, 1.72 mL of 2.31 mM ABTS•+ radical was mixed with 18.28 mL of ethanol, for a final concentration of 200 μM and volume of 20 mL. A volume of 100 μL of 200 μM was added to each well containing 100 μL of the diluted samples. A blank was carried out without a sample. The absorbance was measured at 734 nm, after 20 min of incubation. The formula in Equation (1) was used.

### 4.5. Superoxide Assay Using Alkaline DMSO Method

A modified protocol from Rana et al. [48] was used to perform this experiment with our LT extracts. The superoxide radical was created by using alkaline DMSO. This was created by dissolving 0.250 mL (250 µL) of 1 M NaOH in DI water, to 49.750 uL of DMSO. Reaching an NaOH concentration of 5 mM and volume of 50 mL, the mixture then had air bubbled through it for 1 h. NBT (nitroblue tetrazolium) solution was prepared by dissolving 11 mg of NBT into 11 mL of DI water (pH:7.4), to a final NBT concentration of 1 mg/mL. The sample stock solutions were prepared to a concentration of 4.65 mg/mL. A volume of 43 μL of each sample was added to each well, in order to finish with sample concentration ranging from 1000–0.5 ug/mL. In addition, 143 µL of alkaline DMSO and 14 μL of NBT 1 mg/mL were also added to each well. The plate was incubated for 20 min and read at 560 nm. The final concentrations of the samples ranged from 2–1000 µg/mL, and the final volume in each well was 200 µL. We used DMSO and vitamin-C as negative and positive controls, respectively. The experiment was performed in triplicate.

### 4.6. Iron Reducing Antioxidant Potential (FRAP)

A modified protocol from Dudonne et al. [47] was used to perform this experiment with our LT extracts. A 2 mM stock solution of FeCl_3_ was dissolved in ethanol, by dissolving 16.2 mg of FeCl_3_ into 50 mL of ethanol. A stock solution in ethanolic solvent of 2 mM *O*-Phenanthroline was prepared by dissolving 18 mg of *O*-Phenanthroline into 50 mL of ethanol. 80 μL of FeCl_3_ (2 mM) solution was added to each well. The samples were prepared by dissolving 10 mg of sample in 4 mL of ethanol, to end up with a concentration of 2.5 mg/mL. In addition, 1000 µL of sample (2.5 mg/mL) was placed in test tubes to make a two-fold dilution. The concentrations in the test tubes were as follows: 2.5 mg/mL, 1.25 mg/mL, 0.625 mg/mL, 0.3125 mg/mL, 0.15625 mg/mL, and so on until a final dilution of 4.9 µg/mL. Furthermore, 80 µL of the previous dilutions were placed in the respective wells, with the 80 µL of FeCl_3_. Then, the plate was incubated for 5 min, 40 μL of *O*-Phenanthroline monohydrate was added to each well and read at 510 nm. The final concentration in each well previous to reading ranged from 1000 to 2 μg/mL. We used DMSO and vitamin-C as negative and positive controls, respectively. The experiment was performed in triplicates.

### 4.7. Nitric Oxide (NO) Scavenging Assay Using the Griess Reagent

A modified protocol from Dudonne et al. [47] was used to perform this experiment with our LT extracts. Freshly nitric oxide (NO) radicals were created by the Griess reagent, in which sodium nitroprusside spontaneously generates NO radicals in physiological conditions NO using the Griess reagent is considered a competitive assay, by which 2[NO] reacts with molecular oxygen, making N_2_O_2_, followed by a series of reactions ending with a pink dye. The goal of this assay is to compete with molecular oxygen, arresting the creation of N_2_O_2_. Dilution from 2000–4 μg/mL were prepared by placing 40 μL of samples with an initial concentration of 100 mg/mL diluted in DMSO, and then doing a two-fold dilution. Then, 1.96 mL of DI water was placed in each test tube. Finally, 2 mL of sodium nitroprusside 20 mM in DI water was added to each test tube. Sodium nitroprusside was prepared by mixing 262 mg with 50 mL of DI water. Thus, the final volume was 4 mL, with a concentration of samples ranging from 1000–2 μg/mL, and 10 mM of sodium nitroprusside. This was incubated in the dark at room temperature for 4 h. Then, 180 μL of each test tube was placed in a 96-well plate, and 20 μL of Griess reagent was added. Finally, the samples were incubated in the dark at room temperature for 30 min before reading them at 546 nm. The inhibition properties of the three LT extracts were calculated using the formula shown in Equation (1).

### 4.8. Determination of the Total Phenolic Content

A modified protocol from Ainsworth et al. [4] was used to perform this experiment with our LT extracts. The LT total phenolic content was determined by using the Folin–Ciocalteau colorimetric assay, which utilizes Gallic acid (GA) as a standard reagent. The three LT extract stock solutions were prepared in methanol, with a concentration of 10 mg/mL. GA stock solution was prepared in methanol with a concentration of 5 mg/mL. In order to prepare the standard reference curve using GA, different concentrations of GA were prepared in methanol in the following concentrations: 200, 175, 150, 125, 100, 75, 50 and 25 μg/mL. First, 10% concentration of Folin–Ciocalteu reagent was added to 100 μL of: (1) ethanol LT extract, (2) ethanol: water LT extract, (3) water LT extract, and (4) GA, respectively. Second, the four mixtures were sonicated for 10 s and then incubated for 30 min in the dark. Third, 800 μL of sodium bicarbonate (700 mM in DI water) was added to each LT extract and the control gallic acid, respectively. The four mixtures were further incubated in the dark for 2 h and then read at 765 nm. The final concentration of LT extracts was 500 µg/mL of dry weight. The GA standard curve was calculated by plotting the average absorbance (A765 nm) of the GA concentrations (*y*-axis) versus the GA concentrations (mg/mL; *x*-axis). The GA equivalent was calculated using the following Equation (2):

[(A_s_ − A_b_) − (GA)]/Slope, (2)

where A_s_ is the absorbance of sample, and A_b_ is the absorbance of blank.

### 4.9. Ultraviolet (UV)-Visible Spectroscopy Assay

A modified protocol from [49]. A standard ultraviolet (UV)-visible spectrometer was used to assess the absorption as well as the intensity of each of the LT-extracts at various concentrations. We used the ultraviolet-visible spectroscopy (UV-Vis) to determine the absorbance the 3 LT extracts. Quartz solution cells were used for our control and for each extract.

### 4.10. Cytotoxicity Assay

In order to evaluate the cytotoxicity effect of the three LT extracts, Hs27 fibroblasts (ATCC^®^ CRL-1634^™^) were cultured in 96-well plates at a density of 1 × 10^4^ cells/well at 37 °C in a 5% CO_2_ atmosphere and were treated after one day of incubation. Increased concentrations of each LT extract (3.25–120 µg/mL at 0.25% DMSO highest final concentration) were added to the cells. Then, after 24 h of incubation, Hoechst and Propidium iodide (PI) dyes (5 mg/mL) were added to access the cell viability. The staining pattern resulted from the simultaneous use of these dyes makes it possible to distinguish normal, apoptotic, and dead cell populations by using a cell analyzer (GE Healthcare, Madison, WI, USA). DMSO at 0.25% and H_2_O_2_ 2 mM were used as negative and positive controls, respectively.

### 4.11. High Performance Liquid Chromatography (HPLC) and Mass (MS) Analysis

HPLC. The characterization of the LT ethanol-water extract was carried out using high performance liquid chromatography (HPLC) analysis. The analytical conditions reported by Wong-Paz et al. [50] were followed with slight modifications. Briefly, a Waters 2487 instrument (Waters, Milford, MA, USA) with diode array detection detector set at 280 nm and 330 nm as monitoring wavelength. The separation was achieved on a SunFire (Waters, Milford, MA, USA) C18 5 μm 4.6 × 150 mm column at ambient temperature. The mobile phase consisted of acetonitrile with 1% formic acid (solvent A), water with 1% formic acid (solvent B). The gradient used was: 100% A 0–20 min, 100% B 20–40 min to wash the column. The flow rate was 1 mL/min for 22 min and the injection volume was 10 μL. Fractions were collected, dried and resuspended in HPLC gradient methanol solvent for mass analysis. The identification of NDGA in the LT-ethanol: water extract was based on a combination of retention time and spectral matching. MS Spectrometer. Liquid chromatography/mass spectra (LC-MS) [+ESI] were taken on a double focusing sector type mass spectrometer HX-110A (JEOL Ltd., Tokyo, Japan) (resolution of 10,000 and 10 KV accel. Volt. Ionization method.

### 4.12. Statistical Analysis

Each data point was performed a minimum three times. To indicate experimental variability, data were expressed as the average with corresponding standard deviation. Two-tailed paired Student’s *t*-tests were performed to establish the statistical significance of differences between experimental samples and their corresponding controls. To denote whether comparisons of two treatments have statistical significance, a value of *p* < 0.05 was considered significant. All graphs and IC_50_ values were produced using Graph Pad Prism 7 Software (GraphPad Software, Inc., La Jolla, CA, USA).

## 5. Conclusions

In summary, the HPLC-MS study identified nine natural compounds from the LT-ethanol:water extract that showed antioxidant properties in several in vitro assays without significant cytotoxicity against non-cancerous cell lines. Justicidin B and Beta peltain, which have previously been reported as secondary metabolites of *Larrea tridentata*, were among the identified natural compounds. These natural compounds may possess good pharmaceutical properties and will likely be used as scaffolds for drug discovery investigations. The evaluations of the biological properties related to the antioxidant activity of these natural products are currently under investigation.

## Figures and Tables

**Table 1:** Yield of the *Larrea tridentata* extract in various solvents.

Solvent (*v*:*v*)	LT Dried Leaves (g)	LT Extract (g)	Liquid/Solid	LT Extract Yield (%)
Ethanol	16	3.144	Dark brown oil	19.65
Etanol:Water (60:40)	16	3.964	Dark brown oil	24.78
Water	16	2.258	Bronwnish solid	14.11

**Table 2:** Summary data of the assays performed in this study: FRAP and Total phenolic content assays.

Samples	FRAP ^a^ (250 µg/mL)	Total Phenolic Content (mg GAE/L) ^b^
LT Ethanol	0.950 ± 0.13	200.23 ± 6.25
LT Ethanol:Water	1.01 11 ± 0.12	212.46 ± 7.05
LT Water	0.44 ± 0.06	115.06 ± 10.11
Ascorbic acid	1.035 ± 0.04	Undetermined

^a^ FRAP: Ferric Reducing Antioxidant Power assay; ^b^ GAE/L: Gallic Acid Equivalent/Liters.

**Table 3:** Summary data of the nine collected fractions (F1–F9), their retention time and tentative identified compounds.

LT EtOH/H_2_O Extract HPLC Fractions			
Fraction	Retention Time (min)	Major Fragment Ions *m*/*z* (% Base Peak)	Tentative Identification
F 1	1.592	310.976 (100), 278.976 (80), 121.035 (100), 365.107 (92.17), 172.085 (52.44)	Juglanin ^a^, 2-(4-chlorophenyl)-1-(2,4,6-trihydroxyphenyl)ethenone ^a^, Tyramine ^a^, Justicidin B ^a,b^, Crimidine ^a^
F 2	3.45	365.107 (100), 121.034 (43)	Justicidin B ^a,b^, Tyramine ^a^
F 3	4.051	245.059 (100), 365.110 (94), 413.236 (67), 360.299 (56)	Eleutherol ^a^, Justicidin B ^a,b^, 3′,4′,5,7-Tetraacetoxyflavone ^a^, 3′,4′,5,7-Tetramethylquercetin ^a^
F 4	4.152	365.091 (100)	Justicidin B ^a,b^
F 5	4.308	365.088 (100)	Justicidin B ^a,b^
F 6	4.566	365.084 (100)	Justicidin B ^a,b^
F 7	4.816	365.086 (100)	Justicidin B ^a,b^
F 8	5.183	365.113 (100)	Justicidin B ^a,b^
F 9	5.592	441.272 (100), 365.118 (97), 413.246 (90)	Liquiritin ^a^, Justicidin B ^a,b^, 3′,4′,5,7-Tetraacetoxyflavone ^a^, Podophyllotoxin ^b^, Beta-peltain ^b^

^a^ Confirmed with Mass Spectroscopy (MS) fragmentation and database results. ^b^ Confirmed based on Gnabre et al. [23].

**Table 4:** Summary data of the assays performed in this study: IC_50_ of DPPH, ABTS, Superoxide and NO assays.

Samples	IC_50_ (µg/mL)						
	DPPH ^a^ (1 min)	DPPH ^a^ (10 min)	DPPH ^a^ (20 min)	DPPH ^a^ (30 min)	ABTS ^b^	Superoxide	NO ^c^
LT Ethanol	230.8 ± 3.1	156.3 ± 4.2	143.3 ± 3.5	135.4 ± 3.6	9.75 ± 3.17	2.1 ± 1.2	551.3 ± 112.4
LT Ethanol:Water	209.3 ± 2.2	123.7 ± 4	116.6 ± 3.9	111.7 ± 3.8	8.49 ± 2.28	0.427 ± 0.17	230.4 ± 130.4
LT Water	1471 ± 3.2	866.1 ± 2.8	630.4 ± 4.5	572.7 ± 3.3	35.84 ± 9.19	10.1 ± 6.1	520.7 ± 100.4
Ascorbic acid	49.4 ± 3.9	44.17 ± 1.7	44 ± 4.1	43.9 ± 4	6.82 ± 1.183	12.73 ± 2.75	18.61 ± 6.7

^a^ DPPH: 2,2-diphenyl-1-picryl-hydrazyl-hydrate assay; ^b^ ABTS: (2,2′-Azino-bis(3 ethylbenzthiazoline-6-sulphonic acid)) Radical Scavenging Activity; ^c^ NO: Nitric Oxide Scavenging Assay.
